# Soticlestat, a novel cholesterol 24-hydroxylase inhibitor shows a therapeutic potential for neural hyperexcitation in mice

**DOI:** 10.1038/s41598-020-74036-6

**Published:** 2020-10-13

**Authors:** Toshiya Nishi, Shinichi Kondo, Maki Miyamoto, Sayuri Watanabe, Shigeo Hasegawa, Shigeru Kondo, Jason Yano, Etsurou Watanabe, Tsuyoshi Ishi, Masato Yoshikawa, Haruhi Kamisaki Ando, William Farnaby, Shinji Fujimoto, Eiji Sunahara, Momoko Ohori, Matthew J. During, Takanobu Kuroita, Tatsuki Koike

**Affiliations:** 1 Research, Takeda Pharmaceutical Company Limited, Fujisawa, 251-8555 Japan; 2 Ovid Therapeutics, 1460 Broadway, New York, NY 10036 USA; 3 Takeda Pharmaceutical Company Limited, Cambridge, MA 02139 USA

**Keywords:** Pharmacology, Target validation, Neuroscience

## Abstract

Cholesterol 24-hydroxylase (CH24H) is a brain-specific enzyme that converts cholesterol into 24*S*-hydroxycholesterol, the primary mechanism of cholesterol catabolism in the brain. The therapeutic potential of CH24H activation has been extensively investigated, whereas the effects of CH24H inhibition remain poorly characterized. In this study, the therapeutic potential of CH24H inhibition was investigated using a newly identified small molecule, soticlestat (TAK-935/OV935). The biodistribution and target engagement of soticlestat was assessed in mice. CH24H-knockout mice showed a substantially lower level of soticlestat distribution in the brain than wild-type controls. Furthermore, brain-slice autoradiography studies demonstrated the absence of [^3^H]soticlestat staining in CH24H-knockout mice compared with wild-type mice, indicating a specificity of soticlestat binding to CH24H. The pharmacodynamic effects of soticlestat were characterized in a transgenic mouse model carrying mutated human amyloid precursor protein and presenilin 1 (APP/PS1-Tg). These mice, with excitatory/inhibitory imbalance and short life-span, yielded a remarkable survival benefit when bred with CH24H-knockout animals. Soticlestat lowered brain 24*S*-hydroxycholesterol in a dose-dependent manner and substantially reduced premature deaths of APP/PS1-Tg mice at a dose lowering brain 24*S*-hydroxycholesterol by approximately 50%. Furthermore, microdialysis experiments showed that soticlestat can suppress potassium-evoked extracellular glutamate elevations in the hippocampus. Taken together, these data suggest that soticlestat-mediated inhibition of CH24H may have therapeutic potential for diseases associated with neural hyperexcitation.

## Introduction

The brain is rich in cholesterol, accounting for nearly a quarter of the body’s total cholesterol. Cholesterol plays fundamental roles in maintaining a variety of physiological functions in the brain, such as regulation of membrane potential and release of synaptic vesicles^1–3^. Regulation of cholesterol homeostasis in the brain differs from that in the periphery^4^. One such example is the formation of 24*S*-hydroxycholesterol, which is the dominant pathway of cholesterol catabolism in the brain^5,6^. The excretion of 24*S*-hydroxycholesterol is unidirectional unlike 27-hydroxycholesterol, which can pass through the blood–brain barrier^7^. 24-hydroxylation of cholesterol is catalysed by CYP46A1 (also known as cholesterol 24-hydroxylase [CH24H]), with expression being largely restricted to neurons^8–10^. It is important to note that extracerebral formation of 24*S*-hydroxycholesterol was observed in mice and rats, although the protein expression level of CH24H in liver was less than 1% of that in brain^11^. Considering that 24*S*-hydroxycholesterol is detected in the circulation of CH24H-knockout (KO) mice, 24-hydroxylation of cholesterol can occur through a CH24H-independent mechanism in mice. Nevertheless, in humans, most of the circulating 24*S*-hydroxycholesterol is derived from the brain^12^.

Known as a brain-specific enzyme, CH24H has a potential role in neurological disorders and has been the focus of several genetic studies. For example, investigators have studied the association of polymorphisms of the *CH24H* gene with Alzheimer’s disease (AD)^10,13^ and glaucoma^14^; however, no consensus has been established on the relationship of these polymorphisms with disease. Given that CH24H catalyses the dominant mechanism of the excretion of cholesterol from the brain, its activation has been considered as a therapeutic strategy to stimulate brain cholesterol metabolism. For example, local overexpression of CH24H by a viral vector is beneficial in experimental animal models of AD and Huntington's disease^15–17^. More recently, the reverse transcriptase inhibitor efavirenz has been characterized as a CH24H activator for potential treatment for AD^18–20^. However, such interventional strategies may be limited by the potential toxicity of 24*S*-hydroxycholesterol, the enzymatic product of CH24H. Previously known as ‘cerebrosterol’, 24*S*-hydroxycholesterol has been shown to influence various biological functions, including facilitation of N-methyl-d-aspartate (NMDA) signalling, inflammation, oxidative stress and necroptosis^21–25^, implying that lowering brain 24*S*-hydroxycholesterol levels could also be therapeutic when these factors are driving pathology.

Pharmacological inhibition of CH24H can complement and expand on genetic studies to investigate the therapeutic potential of 24*S*-hydroxycholesterol lowering. Several molecules are known for CH24H inhibitory activity^26^; however, there have been few attempts to test the therapeutic potential of such agents, presumably owing to their non-specificity and pleiotropic activity. One of the earliest examples is voriconazole. This antifungal agent was originally developed as an ergosterol synthesis inhibitor^27^. Although voriconazole reportedly reduces brain 24*S*-hydroxycholesterol, its pharmacological effects are not purely attributable to CH24H inhibition because the drug also interferes with extracerebral cholesterol metabolism. It is, therefore, important to identify a potent, highly-specific and brain-penetrable CH24H inhibitor that merits preclinical and clinical investigations of the effects of perturbating brain cholesterol metabolism. Here, we describe soticlestat ([4-benzyl-4-hydroxypiperidin-1-yl][2,4′-bipyridin-3-yl] methanone, TAK-935, also known as OV935) as a novel CH24H inhibitor. To investigate the pharmacological benefits of soticlestat, we employed a transgenic mouse model carrying human amyloid precursor protein and presenilin 1 (APP/PS1-Tg)^28,29^. Originally developed as an AD model, APP/PS1-Tg is also known for excitatory/inhibitory imbalance and seizure-related sudden death^30,31^. Most importantly, it was shown that cross-breeding of APP/PS1-Tg mice with CH24H-deficient mice extended life-span regardless of zygosity^32^. The observation in conventional knockout mice, however, has left room for the argument that the survival benefit of CH24H insufficiency is attributable to alteration of brain development. Employing a newly identified small-molecule inhibitor, we tested if the high mortality can be reversed by post-development intervention. The present study provides a rationale and supports the hypothesis that pharmacological inhibition of CH24H may have therapeutic relevance to central nervous system (CNS) hyperexcitability.

## Results

### CH24H inhibitory activity and in vitro/in vivo target engagement of soticlestat

**Figure 1:**
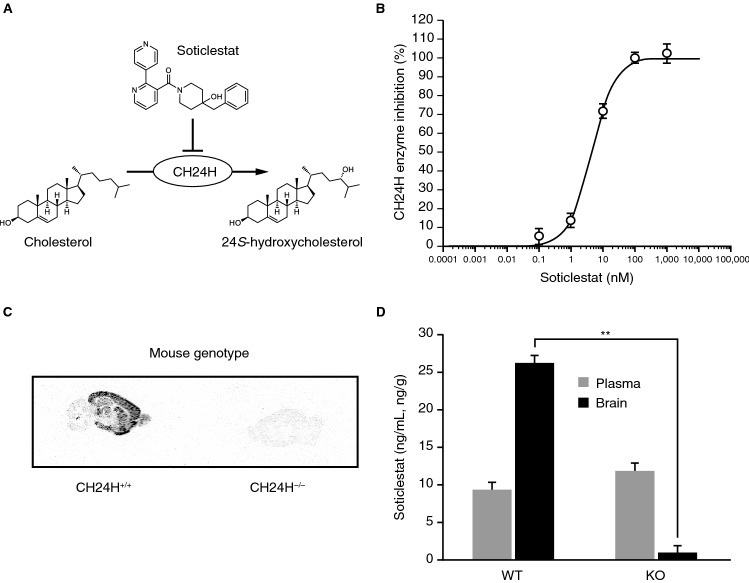
CH24H enzyme inhibition by soticlestat and its target engagement. (**A**) The chemical structure of soticlestat, (4-benzyl-4-hydroxypiperidin-1-yl)(2,4′-bipyridin-3-yl) methanone. (**B**) Human CH24H enzyme inhibition by soticlestat. (**C**) Autoradiographic image of 300 nmol/L [^3^H]soticlestat on brain slices from CH24H-KO and WT control mice (n = 3). Representative images are shown. (**D**) Plasma and brain levels of soticlestat in CH24H-KO and WT mice 1 h after intravenous injection of soticlestat (0.3 mg/kg). Data are mean ± s.e.m. (n = 3). **P < 0.01 (Student’s t-test). CH24H, cholesterol 24-hydroxylase; KO, knockout; s.e.m., standard error of measurement; WT, wild-type.

Soticlestat (Supplementary Fig. S1) was discovered as a result of an iterative medicinal chemistry effort following high-throughput screening of an in vitro enzyme assay (Fig. 1A,B). Soticlestat inhibited the catalytic activity of human CH24H in a concentration-dependent manner with a half-maximal inhibitory concentration (IC_50_) of 4.5 nmol/L. In our preliminary screening assays for major CNS drug targets and drug-metabolizing enzymes, soticlestat did not show notable activities at a concentration 10,000 times higher than the IC_50_ for CH24H inhibition (Supplementary table S1 and S2).

Target engagement specificity was further examined via autoradiography using brain slices collected from wild-type (WT) and CH24H-deficient KO mice (Fig. 1C). The binding of [^3^H]soticlestat (300 nmol/L) showed a clear contrast between WT and CH24H-KO mice. At this concentration, soticlestat inhibitory activity on CH24H is saturated (Fig. 1B). The data suggest that the molecule is highly specific for CH24H. In WT brain slices, the cerebral cortex, thalamus, midbrain and hypothalamus showed relatively stronger binding than the cerebellum, which appeared to be only weakly labelled, implying a relatively broad expression of the enzyme in the cerebrum. The localisation of [^3^H]soticlestat signals agrees with immunohistochemistry of CH24H protein in mouse brain, further supporting the binding specificity to the target (Supplementary Fig. S2A). The lower binding of [^3^H]soticlestat to cerebellum is consistent with our preliminary assessment of regional levels of 24*S*-hydroxycholesterol, which found the highest levels in the striatum and the lowest in the cerebellum (Supplementary Fig. S2B). These observations also agree with a recent study indicating a low level of 24*S*-hydroxycholesterol in the cerebellum^33^, while an earlier immunohistochemistry study detected a relatively high level of CH24H expression in Purkinje cells^34^. To further characterize the CH24H binding specificity of soticlestat, biodistribution experiments were conducted in vivo, comparing CH24H-KO and WT mice following intravenous injection (Fig. 1D). The plasma concentrations of soticlestat 1 h after injection were similar between the two strains at 9.2 ± 4.1 ng/mL and 11.9 ± 2.8 ng/mL (mean ± standard error of measurement [s.e.m.]) for WT and CH24H-KO mice, respectively. In the brain, soticlestat content in WT and CH24H-KO mice was notably different at 26.1 ± 0.8 ng/g and 0.9 ± 0.3 ng/g (mean ± s.e.m.), respectively. These data collectively suggest that soticlestat has an adequately specific affinity to CH24H that deserves further investigation of pharmacodynamics (PD).

### Evaluation of pharmacodynamics (PD) of soticlestat in APP/PS1-Tg mice

**Figure 2:**
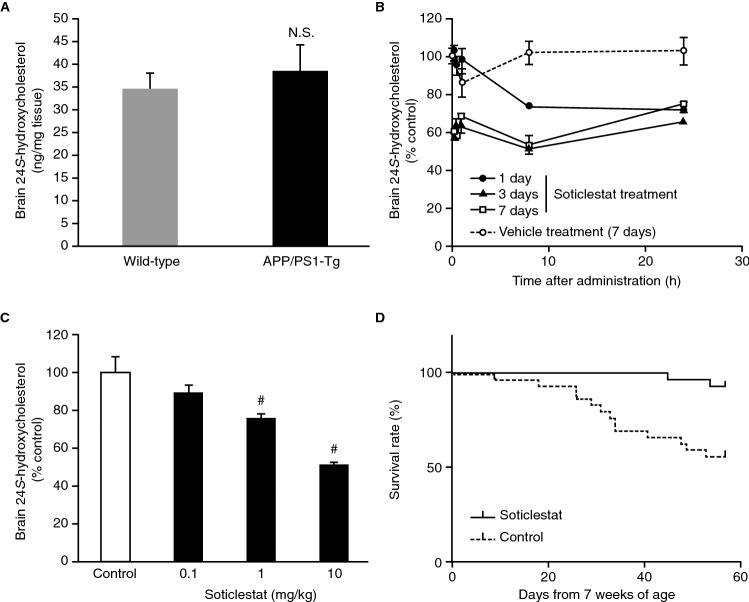
24*S*-hydroxycholesterol lowering effects of soticlestat and survival benefits in APP/PS1-Tg mice. (**A**) Baseline brain 24*S*-hydroxycholesterol levels were compared between APP/PS1-Tg and its WT control at the age of 7 weeks. Data are mean ± s.e.m. (n = 4). (**B**) Time course of brain 24*S*-hydroxycholesterol lowering in APP/PS1-Tg mice by soticlestat treatment for 1, 3 and 7 days (10 mg/kg PO, QD). Vehicle (0.5% methyl cellulose) was administered for 7 days. Data are mean ± s.d. (n = 3). N.S.: not significant (Student’s t-test) (**C**) Dose-dependent reduction of brain 24*S*-hydroxycholesterol in APP/PS1-Tg after 3 days of repetitive soticlestat oral treatment. ^#^*P* < 0.025 (one-tailed Williams test). Data are mean ± s.e.m. (n = 4). (**D**) Kaplan–Meier curves of the survival rate of APP/PS1-Tg mice from 7 weeks of age (n = 30). Mice were treated with vehicle and soticlestat (10 mg/kg PO, QD) over 8 weeks. Statistical significance was assessed by the log-rank test (*P* < 0.001). APP/PS1-Tg, transgenic mouse model carrying mutated human amyloid precursor protein and presenilin 1; PO, orally; QD, once daily; s.d., standard deviation; s.e.m., standard error of measurement; WT, wild-type.

The first study to demonstrate a therapeutic benefit of CH24H inhibition was reported by Halford et al.^32^. In the study, both heterozygous and homozygous CH24H KO remarkably extended the life-span of APP/PS1-Tg mice, while not affecting the amyloid pathology. Based on this study, we employed a strain of APP/PS1-Tg mice with a different PS-1 mutation^28,29^. When brain levels of 24*S*-hydroxycholesterol were evaluated, APP/PS1-Tg mice did not show notably higher levels of brain 24*S*-hydroxycholesterol content than WT animals of the same background strain (Fig. 2A). To test if soticlestat can reduce 24*S*-hydroxycholesterol in the brain, APP/PS1-Tg mice were orally treated with the drug for different time periods as indicated in Fig. 2B. A hysteresis was observed in soticlestat effects on 24*S*-hydroxycholesterol with a temporal pattern that was delayed compared with the pharmacokinetics (PK) data (Fig. 2B and Supplementary Fig. S3A). In a single-dose experiment, soticlestat took 24 h to reach maximum levels of brain 24*S*-hydroxycholesterol lowering (Fig. 2B). Despite a small number of samples, an association was found between PK and PD (Supplementary Fig. S3B, r =  − 0.961). Soticlestat PD effects on brain 24*S*-hydroxycholesterol reached an apparent steady state after repetitive treatments for 3 days. The dose dependency of 24*S*-hydroxycholesterol lowering was further assessed in APP/PS1-Tg mice after 3 days of soticlestat treatment (Fig. 2C). Soticlestat showed a dose-dependent reduction of 24*S*-hydroxycholesterol, reaching a decrease of about 50% at 10 mg/kg. Presumably owing to limited bioavailability in mice, 24*S*-hydroxycholesterol lowering effects of soticlestat almost reached a plateau at the dose of 10 mg/kg and higher with oral gavage (Supplementary Fig. S4). This dose was selected as a treatment condition of soticlestat that yields similar levels of enzyme inhibition to that found in heterozygous CH24H KO^5^. The dose dependency of 24*S*-hydroxycholesterol lowering by soticlestat did not show any noticeable difference in the WT control animals (Supplementary Fig. S5). The potential effects of soticlestat on AD pathology were examined in pilot experiments using 3-month-old APP/PS1-Tg mice. No noticeable effects of soticlestat were observed on amyloid pathology (Supplementary Fig. S6), while a trend was observed on the improvement in working memory deficits in the Y-maze test (Supplementary Fig. S7).

Originally developed as an AD mouse model, APP/PS1-Tg mice have also been recognized as a model of excitatory/inhibitory imbalance, including seizure-related sudden death^30,31,35–37^. The strain kept in our facility showed approximate 50% mortality over the first 3 months after birth. To test the potential effect on survival, soticlestat intervention (10 mg/kg orally [PO], once daily [QD]) was initiated from 7 weeks of age and maintained for 8 weeks. A significant difference was found in the survival curve between the vehicle arm and the soticlestat treatment arm (Fig. 2D). A total of 14 out of 30 mice died in the control group, while 2 out of 30 mice died in the soticlestat group throughout the intervention period. The hazard ratio of death was 5.849 in the vehicle-treated arm compared with the soticlestat arm. The result indicated that soticlestat has a robust pro-survival benefit in the APP/PS1-Tg model in agreement with the earlier study done in CH24H-KO mice^32^.

**Figure 3:**
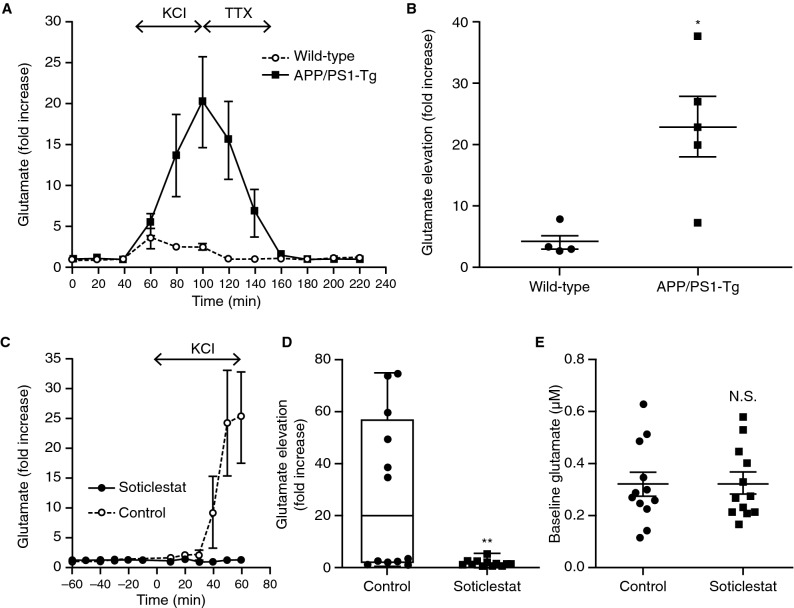
Soticlestat reversed the susceptibility of APP/PS1-Tg mice to potassium-induced glutamate spillover in the hippocampus. (**A**) Elevations in extracellular glutamate in the hippocampus of APP/PS1-Tg and WT mice under 100 mM KCl perfusion, followed by 10 μM TTX. Data are mean ± s.e.m. (n = 4–5). (**B**) The maximum elevations of extracellular glutamate levels during KCl perfusion in the WT and APP/PS1-Tg mice. Data are mean ± s.e.m. (n = 4–5). *P < 0.05 (Student's *t*-test). (**C**) Effects of soticlestat (10 mg/kg PO, QD) on the extracellular level of glutamate before and during hippocampal KCl perfusion following 2-week treatment in APP/PS1-Tg mice. The time when KCl perfusion started was defined as 0 min in the figure. Data are mean ± s.e.m. (n = 12). (**D**) Effects on the elevations in glutamate from baseline for the 60 min KCl perfusion in APP/PS1-Tg mice. The analysis was based on the time-course data shown in (**C**). Data are mean ± s.e.m. (n = 12). **P < 0.01 (Student's *t*-test) (**E**) Effects on the baseline levels of extracellular glutamate collected during the 60 min prior to KCl perfusion. The analysis was based on the time-course data shown in (**C**). Data are mean ± s.e.m. (n = 12). APP/PS1-Tg, transgenic mouse model carrying mutated human amyloid precursor protein and presenilin 1; N.S. not significant; PO, orally; QD, once daily; s.e.m., standard error of measurement; TTX, tetrodotoxin; WT, wild-type.

Interestingly, enhanced cholesterol 24-hydroxylation reportedly decreases astrocytic glutamate sequestration^38^, suggesting a potential role of CH24H in an impaired glutamate uptake system in AD. The APP/PS1-Tg model is also recognized for an epileptic phenotype, as well as for impairment of extracellular potassium homeostasis^30,37^. To unmask the hyperexcitability phenotype, KCl was infused through a microdialysis cannula into the hippocampus of freely moving awake animals^39^. With 100 mM KCl perfused, 7 out of 11 APP/PS1-Tg mice developed lethal seizures, while no obvious behavioural changes were found in WT mice. In pilot experiments, a continuous infusion of KCl led to 100% mortality in APP/PS1-Tg mice unless tetrodotoxin was infused to antagonize the KCl-induced depolarization (Fig. 3A). In contrast, no deaths were observed in WT mice. To control the mortality and to quantify the levels of extracellular glutamate elevation, experiments were terminated after 60 min of KCl perfusion. Glutamate levels in the WT and APP/PS1-Tg mice reached 4.0 ± 1.2 and 23.0 ± 4.9 (mean ± s.e.m.) times baseline levels, respectively (Fig. 3B). The data suggested the susceptibility of APP/PS1-Tg mice to catastrophic depolarization events. To assess the potential effects of soticlestat on the hippocampal hyperexcitability of the model, APP/PS1-Tg mice were treated with soticlestat for 2 weeks (10 mg/kg PO, QD) prior to undergoing microdialysis. It was found that the elevation of extracellular glutamate was greatly suppressed by soticlestat (Fig. 3C,D). Following the initiation of KCl perfusion, seizure-like behaviours became apparent in 50% of the vehicle-treated control mice accompanied by markedly elevated glutamate levels, whereas soticlestat-treated animals showed few behavioural abnormalities. Extracellular glutamate levels peaked at 28.9 ± 8.7 and 1.6 ± 0.4 (mean ± s.e.m.) times baseline levels for control and soticlestat groups, respectively. Meanwhile, soticlestat treatment did not affect baseline glutamate levels before KCl perfusion (Fig. 3E; 0.33 ± 0.04 μmol/L and 0.33 ± 0.04 μmol/L for the control group and soticlestat group, respectively; mean ± s.e.m). In a separate experiment, DL-threo-β-benzyloxyaspartate (TBOA), an inhibitor of astrocytic glutamate uptake, was used to determine the role of glutamate transporters in the observed increase in extracellular glutamate. In the presence of TBOA (10 μmol/L), soticlestat had no effect on extracellular glutamate (Supplementary Fig. S8). It was also reported that glutamate toxicity can induce CH24H translocation from endoplasmic reticulum to the plasma membrane, thereby causing cholesterol loss^40^, which could apply to the APP/PS1-Tg model. However, loss of cholesterol was not found in a detergent resistant membrane component extracted from APP/PS1-Tg brain compared wild-type animals (Supplementary Fig. S9). It was also shown that soticlestat treatment had no considerable impact on the global brain cholesterol levels (Supplementary Fig. S10). Given the intricacy of homeostasis^41^, however, it is possible that impacts of CH24H inhibition of cholesterol levels can vary across different brain regions.

Indiscriminate dampening effects on neural excitation may result in adverse pharmacological phenotypes such as sedation. To support clinical development in terms of preclinical safety, naïve ICR mice were continuously infused with soticlestat for 2 weeks (10 mg/kg, subcutaneous [SC] infusion) to yield a maximum level of 24*S*-hydroxycholesterol lowering and underwent motor phenotype assessments. Under the treatment condition, soticlestat reduced brain 24*S*-hydroxycholesterol by around 96%, while no notable effects on motor coordination and spontaneous locomotor activity were observed (Supplementary Fig. S11). These data collectively suggest that CH24H inhibition can tip the neural excitatory/inhibitory balance without indiscriminately dampening neural excitation.

## Discussion

The brain-specific, cholesterol-catabolic enzyme CH24H has gained increased attention as a potential drug target^18,19^. Clarifying hitherto unproven therapeutic benefits of CH24H inhibitor^26^, the present study provides new insights into the therapeutic relevance of pharmacological modulation of this enzyme^18–20,42^. The data shown here suggest that 24*S*-hydroxycholesterol lowering by soticlestat has therapeutic potential in diseases with underlying excitatory/inhibitory imbalance in the brain. The assessment of soticlestat safety and ‘absorption, distribution, metabolism and excretion’ profile has already led to clinical translation^43^.

To identify a disease condition relevant to CH24H inhibition, we employed the APP/PS1-Tg model, following the previous report that suggested a potential survival benefit of conventional CH24H KO^32^. Chemical inhibition of a protein does not necessarily produce a phenotype aligned with conventional KO of the gene. In this sense, it might be emphasized that soticlestat intervention showed a survival benefit in 7-week-old APP/PS1-Tg mice. One obvious difference from conventional KO is that CH24H was postnatally inhibited by soticlestat in our experiments, indicating that the therapeutic window is not necessarily confined to the prenatal or perinatal period of APP/PS1-Tg mice. It is intriguing to contemplate that activity of cholesterol 24-hydroxylation matters to the balance between life and death depending on the state of brain. Among known mortality factors, neural excitatory/inhibitory balance was highlighted as being important in a recent study of RE1-silencing transcription factor^44^. We are tempted to speculate that the survival benefit of soticlestat is also related to restoration of excitatory/inhibitory imbalance. Soticlestat suppressed the potassium-evoked glutamate elevation in APP/PS1-Tg mice (Fig. 3C,D). Interestingly, its effects on glutamate were almost completely abolished by inhibition of glutamate reuptake by TBOA (Supplementary Fig. S8). Interpretation of the pharmacological experiment with TBOA is challenging; however, the data suggest that CH24H inhibition is likely to affect astrocytic function as well as neuronal function. As mentioned earlier, cholesterol 24-hydroxylation in astrocytes can impair the glutamate reuptake function^38^. Furthermore, Na^+^/K^+^ adenosine triphosphatase activity is impaired in the APP/PS1-Tg model^30^, which is one of the astrocytic risk factors for sudden unexpected death in epilepsy^45^. Given the importance of potassium homeostasis as a risk factor for seizure-related death^46^, the potential involvement of CH24H enzyme activity in the regulation of release and/or clearance of glutamate and/or potassium is an intriguing hypothesis to pursue.

To focus on the mortality-related phenotypes of APP/PS1-Tg mice, soticlestat was characterized mostly in young animals, instead of in aged animals associated with AD-related pathologies, to circumvent a population bias due to the high mortality. Nevertheless, it may be worthwhile briefly describing our preliminary experiments assessing a potential effect of soticlestat on cognitive functions in APP/PS1-Tg mice. One of the reported phenotypes of CH24H-KO mice is an impairment in learning^47^. Conversely, it has been reported that activation of CH24H has pro-cognitive potential^20,48^. Three-month-old APP/PS1-Tg animals were treated with soticlestat and underwent the Y-maze test. Although a pharmacological experiment in 3-month-old APP/PS1-Tg mice does include a survival bias, our preliminary data, to the contrary, showed an apparent trend on the improvement in working memory (Supplementary Fig. S7). This observation may disagree with the cognitive benefits of efavirenz, a reverse transcriptase inhibitor known for CH24H activation^20,48^. It should be noted that the effects of efavirenz on cognition are apparently associated with improvement in amyloid pathology. In our pilot experiments, however, soticlestat treatment had no notable impact on the amyloid pathology of APP/PS1-Tg mice (Supplementary Fig. S6). The lack of effects on amyloid pathology is also consistent with the phenotype of CH24H KO^32^. The improving trend of cognition seen in soticlestat could be explained by other mechanisms such as modulation of the excitatory/inhibitory balance.

One of the rationales for pharmacological activation of CH24H is acceleration of brain cholesterol turn-over, which is expected to be beneficial for some neurodegenerative diseases^15,20,49^; however, this hypothesis may not be readily generalizable to other diseases. Firstly, acceleration of cholesterol 24-hydroxylation can disrupt the integrity of membrane^50^, thereby possibly disturbing physiological functions of neurons^1–3^. Secondly, CH24H activation leads to upregulation of cholesterol biosynthesis, an energy-demanding process that consumes 18 mol of acetyl coenzyme A, 18 mol of adenosine triphosphate and 29 mol of nicotinamide adenine dinucleotide phosphate to produce 1 mol of cholesterol^51^. As the brain is an organ of high-energy demand and 100% self-sufficiency in cholesterol production, activation of this costly mechanism may add an extra burden on the brain^52^. Further studies are needed to understand whether activation or inhibition of CH24H can be therapeutic for each disease of interest. Future studies are planned to clarify whether or not, and how CH24H inhibition can modulate cholesterol homeostasis in the brain. We propose that 24*S*-hydroxycholesterol lowering can be a therapeutic approach when this oxysterol is driving such pathology as inflammation, oxidative stress and excitatory/inhibitory imbalance^24,25,53^. Soticlestat is being tested in clinical trials for the treatment of epilepsy. In a separate study, soticlestat has been shown to ameliorate seizure progression in correlation with lowering of brain 24*S*-hydroxycholesterol in a mouse model of epilepsy (r = − 0.682; *P* < 0.05, Pearson correlation coefficient)^43^. The full set of data will be published elsewhere.

Finally, it is interesting to note that CH24H knockdown can be neurotoxic in other animal models^16,54^. The hypothesis is, however, based on a viral vector-mediated RNA interference introduced locally in the hippocampus. Given the intricate interrelationship of neural circuits, it is possible that a regional somatic cell CH24H knockdown, with uncertain specificity and using viral vectors with a known neuronal tropism, produces a result different from a pharmacological inhibition of the enzyme across all cell types and in the entire brain. Importantly, a nearly full inhibition of CH24H by soticlestat did not cause notable abnormalities in motor coordination and spontaneous locomotor activities (Supplementary Fig. S11). In fact, hyperlocomotion is a typical behavioural phenotype of NMDA receptor (NMDAR) blocker^55^. Reduction of brain 24*S*-hydroxycholesterol, known as a potentiator NMDAR, may produce a similar phenotype, but this was not the case in our observation. As a matter of fact, the brain level of 24*S*-hydroxycholesterol is estimated at around 25 μM across mammalian species, 20-fold higher than the reported half-maximal effective concentration on NMDAR^21^. Therefore, it is not surprising that even aggressive CH24H inhibition by soticlestat does not disturb the baseline NMDAR functions.

In summary, the experiments conducted in this study collectively demonstrate the therapeutic potential of soticlestat. Considering the in vitro and in vivo assessment of soticlestat target engagement, it can be concluded that CH24H inhibitor has a therapeutic potential that merits further investigation, not only in the clinical setting, but also as a basic research tool to shed light on the biological implications of 24*S*-hydroxycholesterol.

## Methods

### Measurement of CH24H inhibitory activity

CH24H enzyme was expressed by transducing the full-length *CH24H* gene (NCBI Accession Number BC022539) into FreeStyle 293-F cells (Invitrogen, Carlsbad, CA, USA). A CH24H lysate product was prepared from supernatant isolated by centrifugation of the homogenate. Catalytic activity of the CH24H enzyme was measured using thin-layer chromatography (TLC). To evaluate the inhibitory activity, 2 μL of serial diluted compounds were incubated with 3 μL of CH24H enzyme in assay buffer (50 mM potassium phosphate buffer [pH 7.4]) supplemented with 0.1% bovine serum albumin (BSA) and cOmplete EDTA-free tablets (Roche, Basel, Switzerland) for about 15 min at room temperature. The final concentration of dimethyl sulfoxide (DMSO) in the assay was 0.2% when the compound was tested in duplicate in 384-well plates. The reaction was started with the addition of 5 μL of substrate [^14^C]cholesterol (PerkinElmer, Foster City, CA, USA, NEC018250UC) at a final concentration of 15 μM, which was dissolved in assay buffer containing 2 mM β-NADPH. After 5 h of incubation at 37 °C, 35 μL of chloroform:ethanol (1:2 v/v) was added to terminate the CH24H reaction. After mixing, 25 μL of distilled water containing 0.0024% trypan blue was added to the mixture. Based on the Bligh and Dyer total lipid extraction method, 4.5 μL of the lower layer was spotted on the TLC plate (Silica Gel 60 F_254,_ Merck, Darmstadt, Germany). The TLC plates were developed using ethyl acetate:toluene (2:3 v/v) and visualized by the ImageQuant TL software version 8.1 (GE Healthcare Ltd, Chalfont St Giles, UK) and analysed by the Multi Gauge software version 2.3 (Fujifilm Corporation, Tokyo, Japan). The band at Rf 0.53 was identified as 24*S*-hydroxycholesterol by LC/MS/MS and quantified by the analyser.

### In vitro autoradiography in CH24H WT and KO mouse brain sections

Twenty-seven-week-old male CH24H WT and KO mice were euthanized by decapitation. The brains were rapidly removed and slowly frozen by immersion into an isopentane–dry ice bath. The frozen brains were stored in a deep freezer. Twenty micrometre-thick sagittal or coronal sections were cut on a cryostat (Leica Microsystems, Wetzlar, Germany) and thaw-mounted onto glass slides. The mouse sagittal sections were prepared from within 1.1–1.2 mm lateral to the midline (coordinates taken from the Franklin and Paxinos mouse brain atlas^56^). Sagittal sections prepared from CH24H WT and KO mouse brains (3 mice in each genotype) on glass slides were warmed to room temperature. The sections were preincubated twice for 5 min at room temperature in preincubation buffer (50 mmol/L of Tris–HCl pH 7.5, 1.7 mmol/L of EDTA, 6 mmol/L of MgCl_2_, 120 mmol/L of NaCl, and 0.1% BSA). The sections were treated with 300 nmol/L of [^3^H]soticlestat in binding buffer (preincubation buffer containing 0.03% Triton X-100) for 4 h at room temperature. The sections were rinsed twice for 5 min at 4 °C in preincubation buffer and then rapidly rinsed in ice-cold distilled water. The sections were dried overnight at room temperature and exposed to imaging plates BAS-IP TR 2040 E (GE Healthcare Ltd, Chalfont St Giles, UK) for 7 days. The imaging plates were analysed using an image analyser FLA7000 (Fujifilm, Tokyo, Japan) and the ImageQuant TL software version 8.1 (GE Healthcare Ltd, Chalfont St Giles, UK).

### Preparation of soticlestat

For oral administration, soticlestat was suspended in 0.5% (w/v) methyl cellulose solution (Methyl Cellulose 50cp, Wako Pure Chemical Industries, Tokyo, Japan), and the prepared drug suspension was administered QD to mice at the volume of 10 mL/kg. SC infusion was employed to maximise the pharmacodynamics of soticlestat. Soticlestat was dissolved in DMSO and mixed with an equal volume of polyethylene glycol 400 (PEG 400). Osmotic pumps (Model 2004, Durect, Cupertino, CA, USA) filled with the prepared drug solution were implanted SC on the backs of the mice under anaesthesia.

### Measurement of soticlestat exposure levels in plasma and brain

Plasma and brain were collected at the indicated time points to determine the exposure level of soticlestat following single and chronic administration. Whole blood was collected from the inferior vena cava with heparin. To collect brain samples, cerebellum was removed. The collected brain was homogenized with saline for subsequent procedures. The aliquots of plasma and the brain homogenate were mixed with acetonitrile containing deuterated soticlestat analogue as internal standard and then centrifuged. The supernatants were diluted with solvents for liquid chromatography–tandem mass spectrometry (LC–MS/MS) (mobile phase A: 10 mM of ammonium formate:formic acid [100:0.2 v/v]; mobile phase B: acetonitrile:formic acid [100:0.2 v/v]). The diluted solutions (1–10 μL) were injected into LC–MS/MS solvent (API 5000, AB SCIEX, Foster City, CA, USA) that was equipped with Shimadzu Shim-pack XR-ODS (2.2 μm, 2.0 × 30 mm) (Shimadzu, Kyoto, Japan) maintained at 50 °C. The chromatographic separation was performed with a gradient elution containing two mobile phases, A and B, at a flow rate of 0.7 mL/min. Mobile phase B was held at 5% for 0.2 min and increased linearly to 99% for 1.1 min. After phase B was held at 99% for another 0.7 min, it was brought back to 5% in 0.01 min, followed by re-equilibration for 0.59 min. The total cycle time for one injection was 2.6 min. Compounds were detected using a multiple reaction monitoring mode using this transition: soticlestat m/z 374.3 ([4-benzyl-4-hydroxypiperidin-1-yl][2,4′-bipyridin-3-yl] methanone) → 155.2 /183.1 ([2,4′-bipyridin-3-yl]methanone moiety, Supplementary Fig. S12), soticlestat analogue m/z 379.4 → 155.2 /183.1 ([2,4′-bipyridin-3-yl]methanone moiety). Analyst Software version 1.4.2 (SCIEX, Redwood City, CA, USA) was used for data acquisition and processing. The lower limit of quantification of soticlestat was 0.1 ng/mL and 0.5 ng/g in plasma and brain, respectively.

### Measurement of 24*S*-hydroxycholesterol levels in brain

In this study, high-performance liquid chromatography (HPLC)-based assay was employed to improve the throughput of drug discovery campaign instead of LC–MS/MS analysis, which is used for plasma 24*S*-hydroxycholesterol analysis in clinical trials^57^. To determine the level of 24*S*-hydroxycholesterol in the brain, brain homogenate was prepared as described above. 24*S*-hydroxycholesterol was extracted from brain homogenate by mixing with equal volume (w/v) of 98% acetonitrile containing 2% methanol and 0.2% formic acid, and then centrifuged at 12,000*g* for 15 min at room temperature. The supernatant was filtrated with a 0.45 μm filter and measured by high-performance liquid chromatography (HPLC). A ready-to-use prepacked C18 column (Capcell Pak C18 MGII, 3 µm, 3.0 mm inner diameter [ID] × 75 mm length, Shiseido, Kyoto, Japan) with a precolumn packed with the same material (silica gel) was used for separation. Diluted water with 0.1% trifluoroacetic acid (A) and methanol with 0.1% trifluoroacetic acid (B) were used for the chromatographic run. The composition of the mobile phase was changed according to the following time programme: 0–1 min 20% (A) and 80% (B); 1–21 min 10% (A) and 90% (B); 21–25 min 100% (B); and 25–30 min 100% (B). The flow rate was 0.5 mL/min. Peaks were detected by an ultraviolet (UV) detector at 210 nm. The peak at the retention time of 13 min was extracted by fraction collector and confirmed as 24*S*-hydroxycholesterol by LC–MS/MS. The peak area was analysed by using the LabSolutions software version 5.57 (Shimadzu, Kyoto, Japan). The HPLC method estimate the baseline 24*S*-hydroxycholesterol at 30 µg/mg tissue, which roughly agree with that reported in literature. The 24*S*-hydroxycholesterol peak was not detected from brain samples derived from CH24H-KO mice, suggesting that the HPLC condition sufficiently separate 24*S*-hydroxycholesterol from other contaminants that would undermine the quantification (Supplementary Fig. S13).

### Animal models

CH24H-KO mice were purchased (B6. 129S7-Cyp46a1Rus/J, Jackson Laboratory, Sacramento, CA, USA). APP/PS1-Tg mice were obtained by cross-breeding APP-SW mice (Tg2576)^28^ with PS-1M146L knock-in mice^29^. The female mice were maintained in house and used for the survival experiment at 7 weeks of age. The animals were housed at a temperature of 22 °C ± 1 °C (mean ± s.d.) with a 12 h light–dark cycle (lights on from 07:00 to 19:00) and allowed free access to food and water.

### Survival observation of APP/PS1-Tg mice

The survival of 7-week-old APP/PS1-Tg mice was monitored for 8 weeks. Mice were orally treated with vehicle (0.5% methyl cellulose, n = 30) or soticlestat at the dose of 10 mg/kg (n = 30) QD until they died.

### Glutamate microdialysis

The APP/PS1-Tg mice treated with vehicle (0.5% methyl cellulose; n = 12) and those treated with soticlestat (n = 12) were subjected to the microdialysis study. Under pentobarbital-chlorohydrate anaesthesia, the guide cannula (AMUZA Inc, San Diego, CA, USA) was stereotactically implanted in the left hippocampus at the following coordinates (mm from bregma): A–3.1, L + 2.8, V–2.0. One week after the surgery, a microdialysis probe (depth 4 mm, length 2 mm) was inserted through the guide cannula, and the hippocampus was continuously perfused with Ringer’s solution (147 mmol/L of NaCl, 4.03 mmol/L of KCl and 0.30 mmol/L of CaCl_2_) at a rate of 2 µL/min for 3 h. The total sampling time was 130 min, consisting of 13 samplings (10 min intervals each). The first sample (0–10 min) was not used for analysis because of injection noise. The next six samples (10–70 min) were collected as a baseline. The perfusion fluid was then switched from normal Ringer’s solution to the Ringer’s solution containing 100 mmol/L of KCl with six subsequent samples (70–130 min) being collected. Analysis of dialysis samples was performed by reversed-phase HPLC operated by the PowerChrome software version 2.5.8 (eDAQ, Denistone East, Australia). The baseline glutamate level was calculated as an average concentration in the first 60 min. The potassium-induced glutamate elevation was defined as the ratio of the maximum value to the baseline average.

### Study approval

The animal experiments were approved by the Experimental Animal Care and Use Committee of Takeda Pharmaceutical Company Ltd and conducted in accordance with the guidelines. The animal care and use programme is accredited by the American Association for Accreditation of Laboratory Animal Care (AAALAC) International’s Council on Accreditation. The AAALAC sets standards that call for the humane care and use of laboratory animals by enhancing animal well-being, improving the quality of research and advancing scientific knowledge relevant to humans and animals.

### Statistical analysis

The statistical significance between two experimental groups was evaluated by Student’s t-test, with probability values (*P*) less than 5% considered significant (*P < 0.05, **P < 0.01). To evaluate dose dependency, one-tailed Williams’ test was used and probability values less than 2.5% were considered significant (^#^*P* < 0.025).

## Supplementary information

Supplementary Information.

## Data Availability

The data that support the findings of this study are available on request from the corresponding author (T.N.). The data are not publicly available due to Takeda Pharmaceuticals publication policy.
