# Supplementary material for: Soticlestat, a novel cholesterol 24-hydroxylase inhibitor shows a therapeutic potential for neural hyperexcitation in mice
Source: Sci Rep. 2020 Oct 13;10:17081. doi: 10.1038/s41598-020-74036-6 (PMC7553946; doi:10.1038/s41598-020-74036-6)
Supplement: Supplementary file 1 — Supplementary Information. [file 41598_2020_74036_MOESM1_ESM.pdf]

Supplementary information

Soticlestat, a novel cholesterol 24-hydroxylase inhibitor shows a therapeutic potential for neural hyperexcitation in mice

Authors

Toshiya Nishi[1]*, Shinichi Kondo[1], Maki Miyamoto[1], Sayuri Watanabe[1], Shigeo Hasegawa[1], Shigeru Kondo[1], Jason Yano[1], Etsurou Watanabe[1], Tsuyoshi Ishi[1], Masato Yoshikawa[1], Haruhi Kamisaki Ando[1], William Farnaby[1], Shinji Fujimoto[1], Eiji Sunahara[1], Momoko Ohori[1], Matthew J. During[2], Takanobu Kuroita[1] & Tatsuki Koike[1]

[1]Research, Takeda Pharmaceutical Company Limited, Fujisawa 251-8555, Japan.

[2]Ovid Therapeutics, 1460 Broadway, New York, NY 10036, USA.

***Corresponding author:**

Toshiya Nishi, Takeda Pharmaceutical Company Limited, Cambridge, 02139 MA, USA, Tel: +1-617-551-8786, toshiya.nishi@takeda.com

Supplementary figures

1H NMR

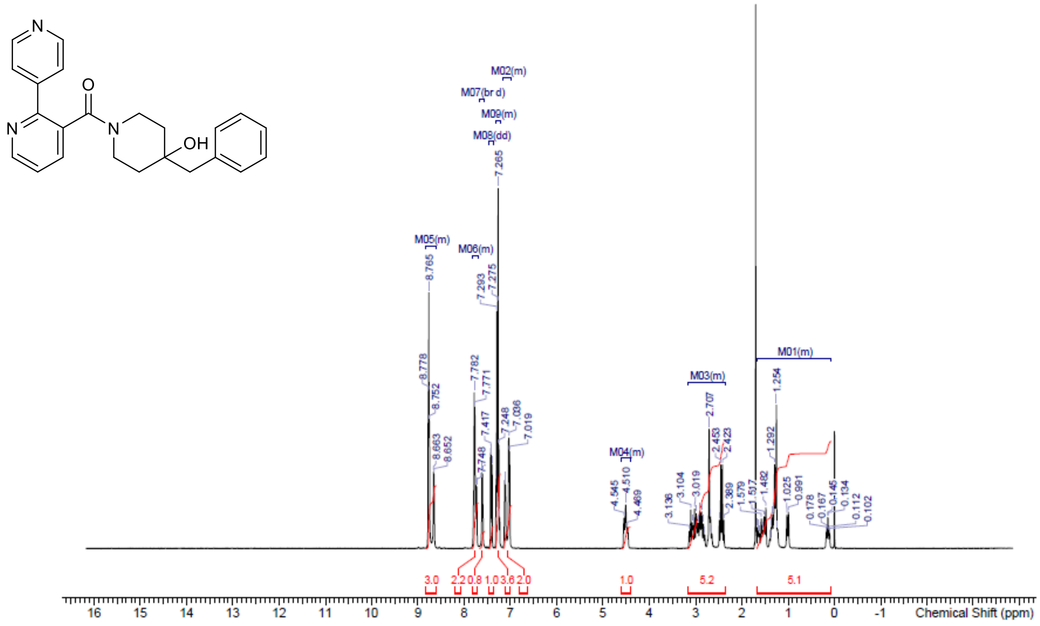

13C NMR

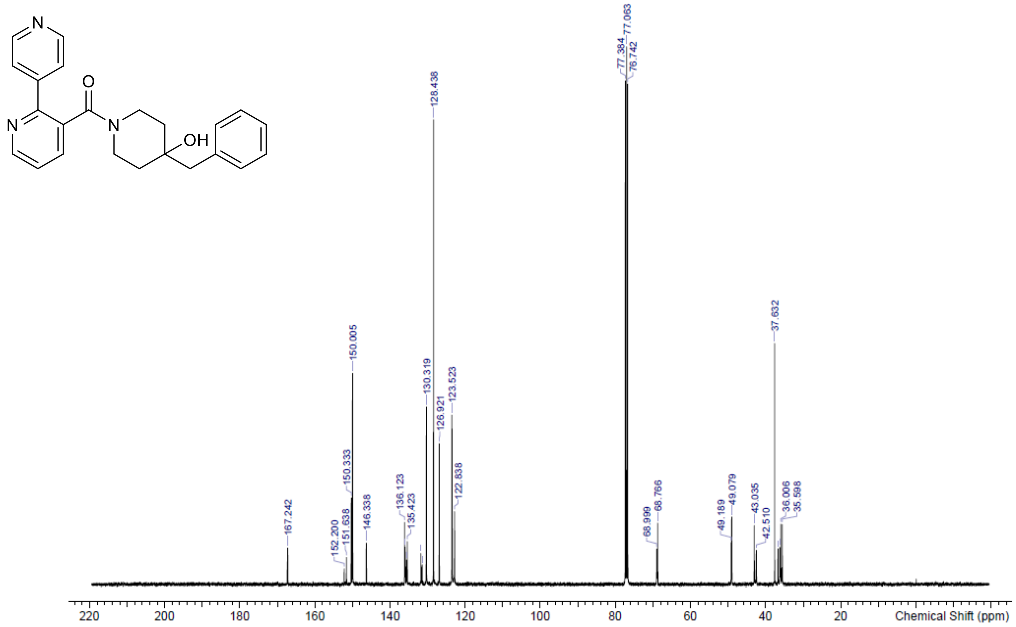

Supplementary figure S1. NMR spectroscopic analysis of soticlestat. NMR, nuclear magnetic resonance; ppm, parts per million.

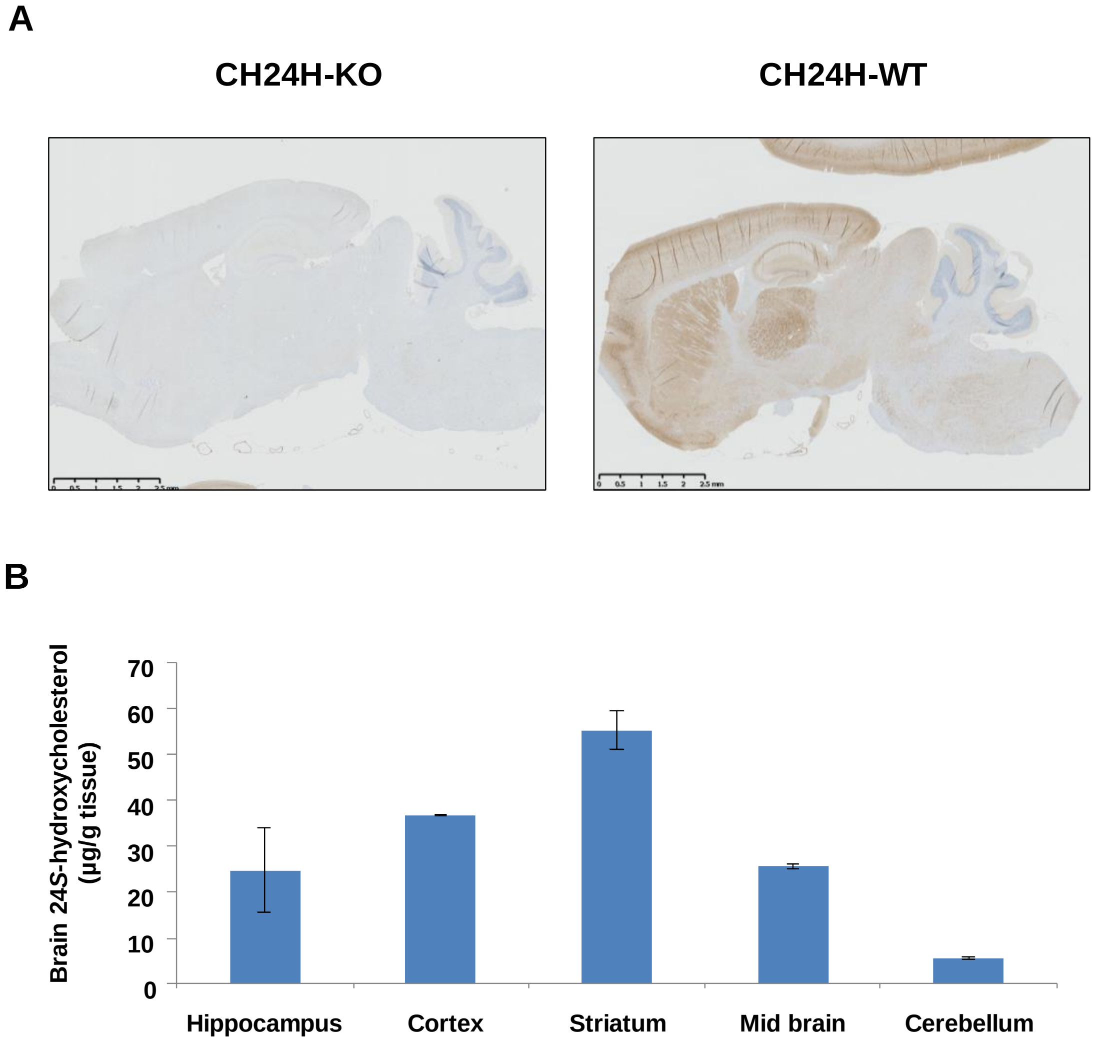

Supplementary figure S2. (**A**) Representative CH24H immunohistochemistry images in comparison between CH24H-KO and -WT mouse brain sagittal sections. Four µm sagittal paraffin section slides were stained by CYP46A1 rabbit polyclonal antibody (Proteintech Cat.# 12486-1-AP, 6.7µg/mL final concentration). The staining was conducted automatically by Roche VENTANA Discovery auto-staining system. The Extended CC1 protocol was employed for antigen retrieval. A goat anti-rabbit IgG antibody was used as the 2nd antibody (Vector Cat.# BA-1000, 5µg/mL final concentration). Hematoxylin was used as a counter staining. (**B**) Brain 24*S*-hydroxycholesterol contents in the hippocampus, cortex, striatum, midbrain and cerebellum of neurologically healthy mice. Data are mean ± s.d. (n = 4). CH24H, cholesterol 24-hydroxylase; WT, wild type; KO, knock out; s.d., standard deviation.

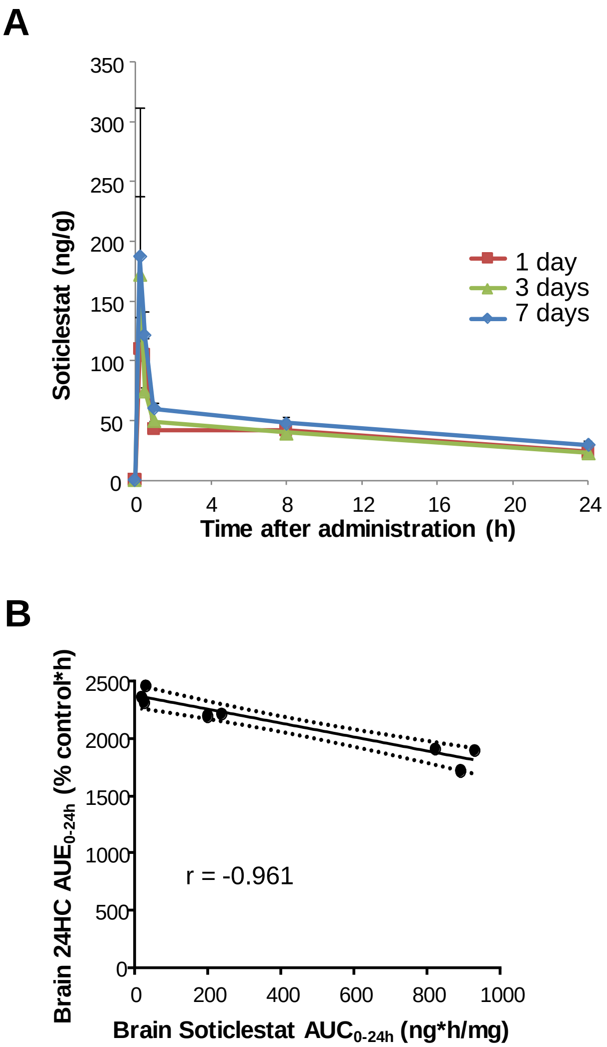

Supplementary figure S3. (**A**) Time course of brain levels of soticlestat at 0.25, 0.5, 1, 8 and 24 h following the indicated dosing protocol (10 mg/kg PO, QD). Data are mean ± s.d. (n = 3). (**B**) PK/PD correlation was evaluated after a single administration of soticlestat (n = 3 for each of 0.1, 1 and 10 mg/kg PO). The PD effect was evaluated as the area under the curve of brain 24HC levels for 24 h ($AUE_{0\text{-}24h}$). The exposure was shown as the area under the curve of brain concentrations of soticlestat for the matched time frame ($AUC_{0\text{-}24h}$). The dashed lines define the 95% confidence band in linear regression ($P < 0.01$, Pearson's test). 24HC, 24*S*-hydroxycholesterol; PD, pharmacodynamics; PK, pharmacokinetics; PO, orally; QD, once daily; s.d., standard deviation.

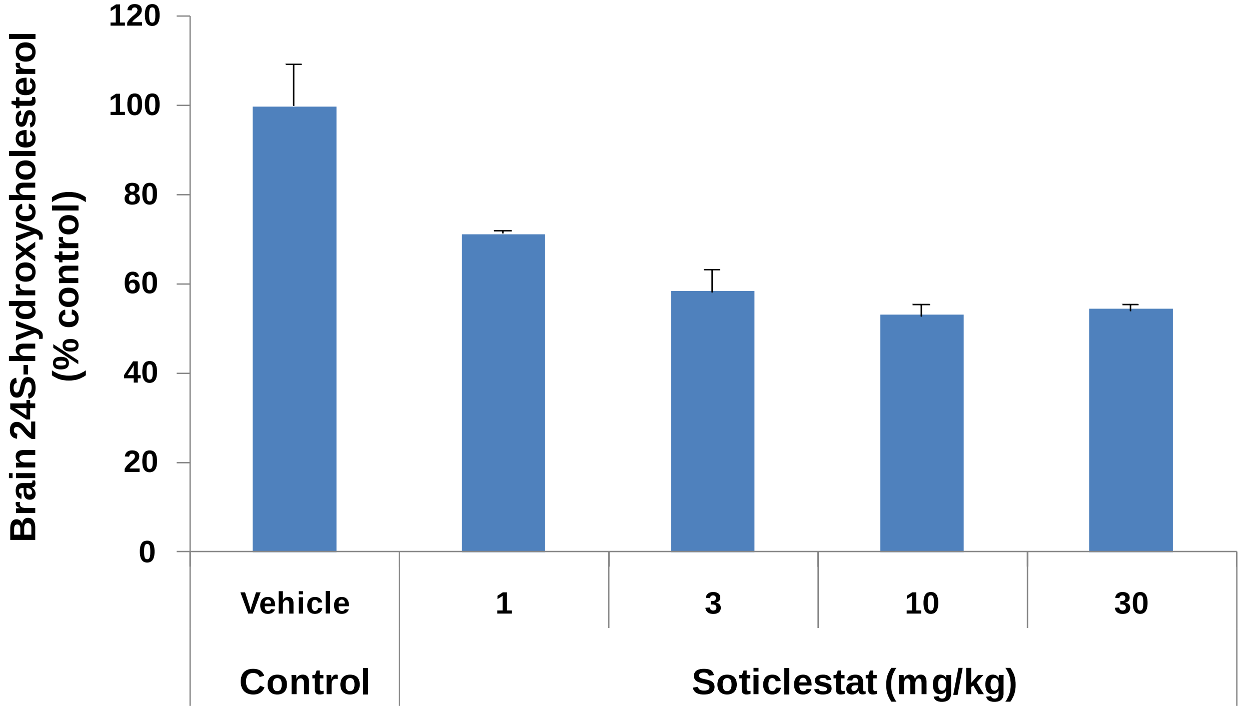

Supplementary figure S4. The dose-dependent effects of soticlestat on brain 24*S*-hydroxycholesterol levels in neurologically healthy mice. Data are mean ± s.d. (n = 4). ctrl, control; s.d., standard deviation.

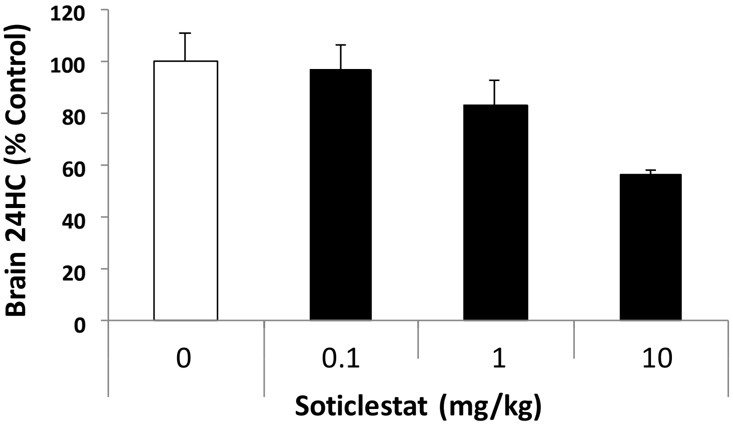

Supplementary figure S5. The dose-dependent effects of soticlestat on brain 24HC levels in WT control mice of APP/PS1-Tg. Data are mean ± s.d. (n = 4). 24HC, 24*S*-hydroxycholesterol; APP/PS1-Tg, transgenic mouse model carrying mutated human amyloid precursor protein and presenilin 1; s.d., standard deviation; WT, wild-type.

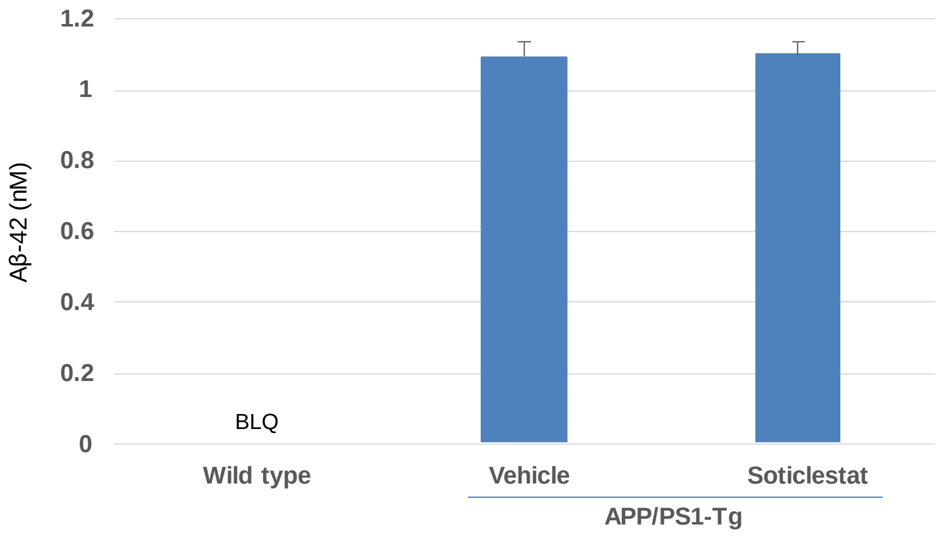

Supplementary figure S6. The concentration of human amyloid-beta 42 (Aβ-42) in the hippocampus of APP/PS1-Tg mice. Levels in wild-type animals were below the lower limit of quantification (BLQ). Three-month-old APP/PS1-Tg mice were treated with either vehicle or soticlestat (10 mg/kg PO, QD) for 2 weeks. No statistical significance was detected between the vehicle and soticlestat group (Student's t-test). Data are mean ± s.e.m. (n=8). APP/PS1-Tg, transgenic mouse model carrying mutated human amyloid precursor protein and presenilin 1; PO, orally; QD, once daily; s.e.m, standard error of measurement.

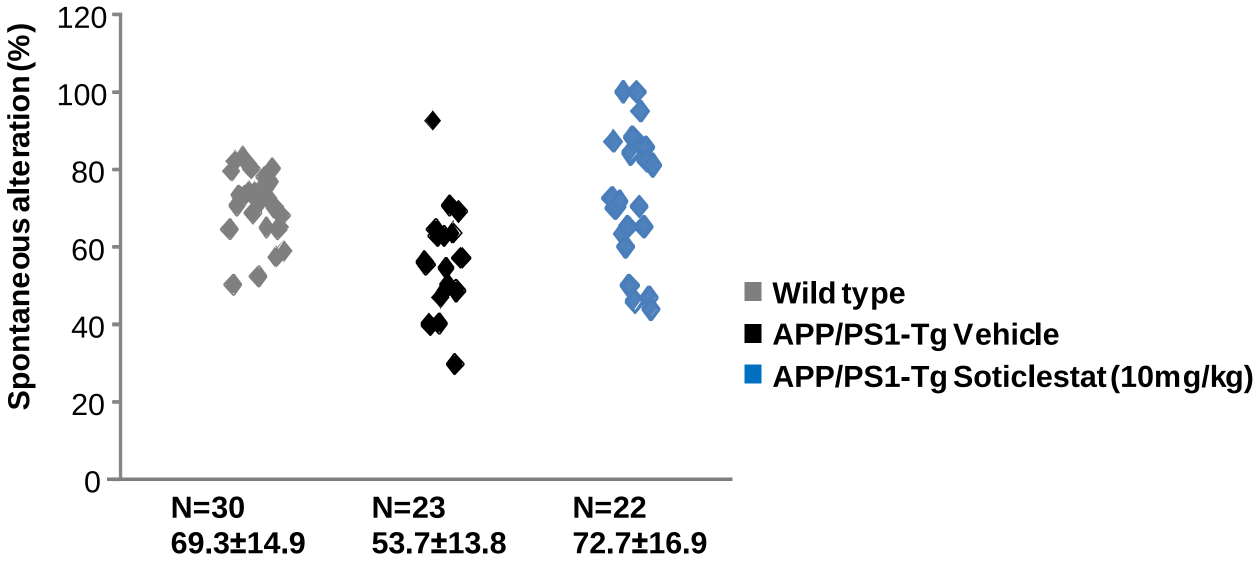

Supplementary figure S7. The Y-maze performance of 3-month-old APP/PS1-Tg and WT mice. Soticlestat (10 mg/kg PO, QD) was administered for 2 weeks before the behavioural assessment. Data are mean ± s.d. APP/PS1-Tg, transgenic mouse model carrying mutated human amyloid precursor protein and presenilin 1; PO, orally; QD, once daily; s.d., standard deviation; WT, wild-type.

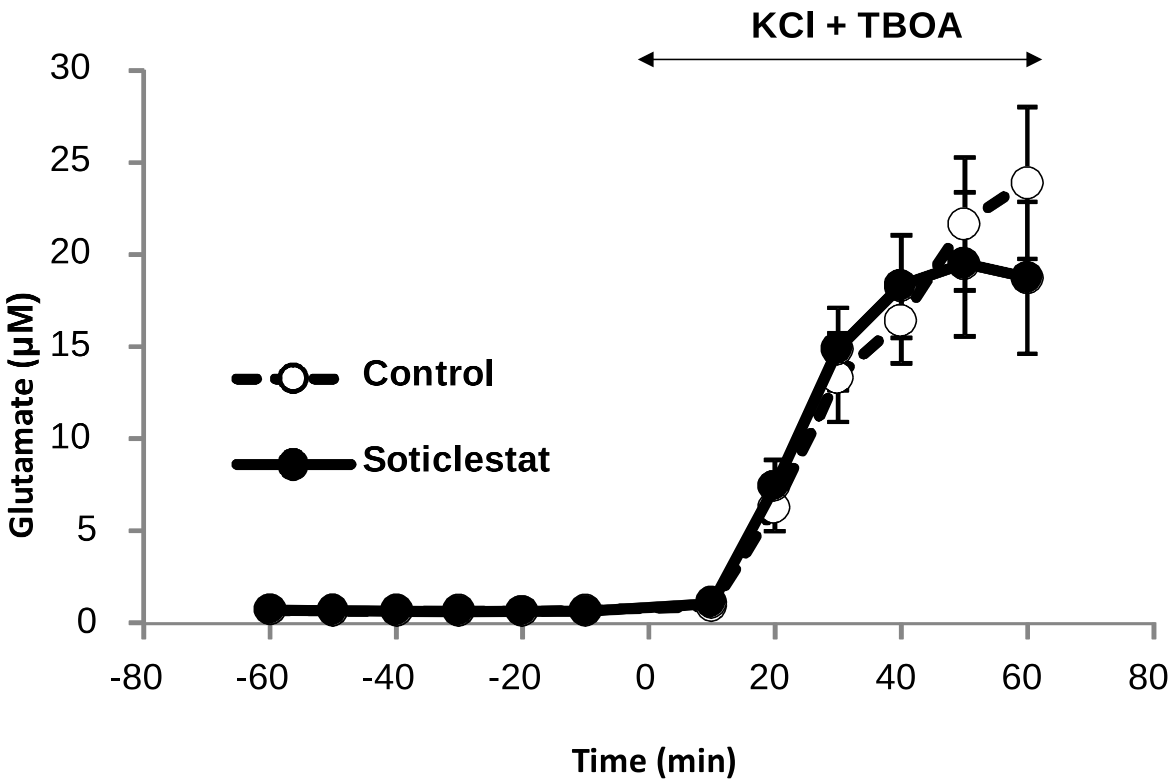

Supplementary figure S8. Effects of TBOA on KCl-evoked extracellular glutamate elevations in the hippocampus of APP/PS1-Tg mice. Soticlestat was administered for 2 weeks before microdialysis experiments (10 mg/kg PO, QD). The time when KCl (100 mM) and TBOA (10 µM) perfusion started was defined as 0 min in the figure. Data are mean ± s.e.m. (n = 10). APP/PS1-Tg, transgenic mouse model carrying mutated human amyloid precursor protein and presenilin 1; PO, orally; QD, once daily; s.e.m., standard error of measurement; TBOA, DL-threo-β-benzyloxyaspartate.

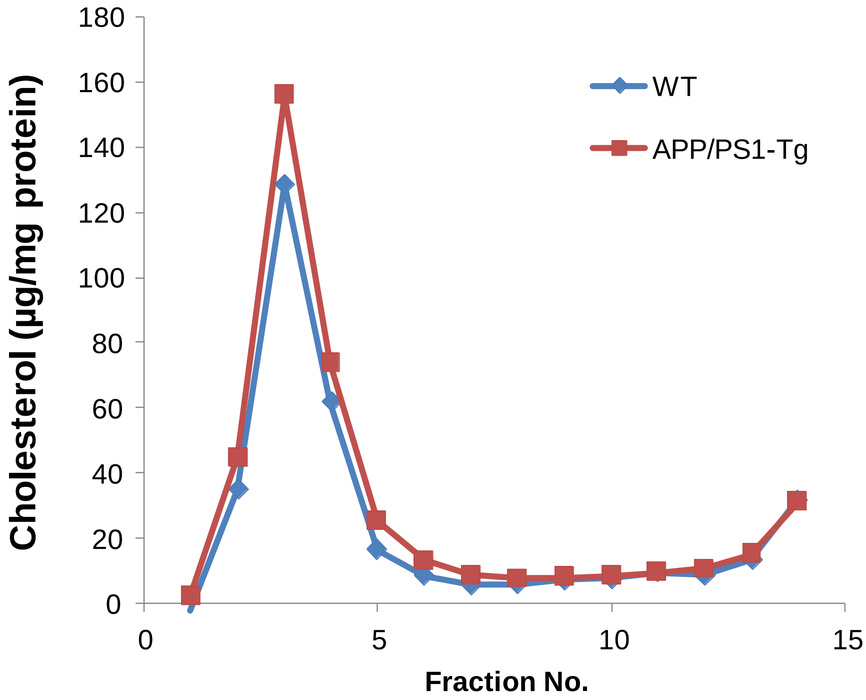

Supplementary figure S9. Cholesterol contents in the membrane fractions of APP/PS1-Tg and WT mice. To induce a glutamate toxicity insult, 100 mM of KCl was continuously injected into the hippocampus. Brain samples were collected 60 min after KCl perfusion was initiated. Following myelin removal treatment (Myelin removal beads, Miltenyi), a crude membrane fraction was treated with 3% Brij58 for 1 hour on ice. The detergent-treated sample underwent centrifugation on a discontinuous 5%/35%/42.5% sucrose density gradient. Fractions 3 to 5 were considered as the detergent-resistant floating low-density fractions. Cholesterol concentration of each fraction was determined by Amplex Red Cholesterol Assay Kit (Molecular Probes). Cholesterol levels were normalised by protein concentrations determined by the BCA method (Pierce). APP/PS1-Tg, transgenic mouse model carrying mutated human amyloid precursor protein and presenilin 1; WT, wild-type.

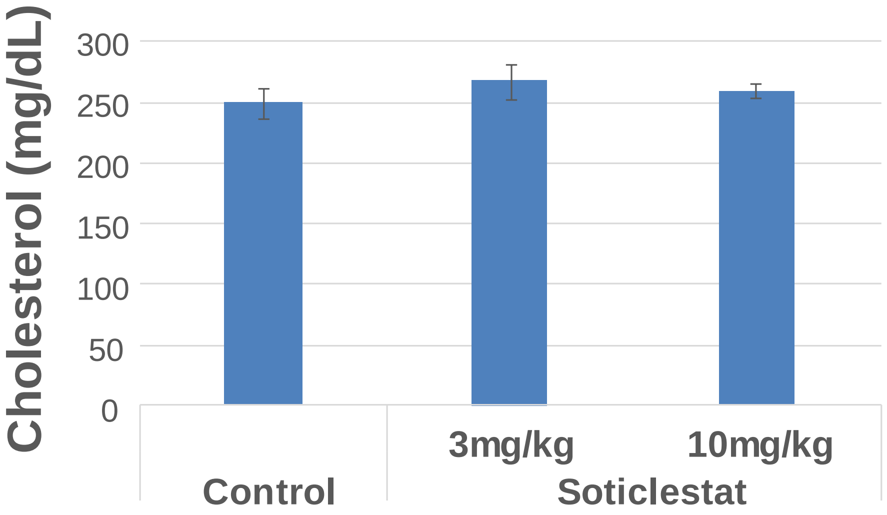

Supplementary figure S10. Effects of soticlestat treatment on brain levels of total cholesterol in APP/PS1-Tg. Mice were treated with soticlestat for 2 weeks at the indicated doses. Homogenate was prepared from the brain after removal of cerebellum due to little CH24H expression (Fig. S2). The homogenate underwent liquid-liquid phase separation with n-hexane/isopropyl alcohol (60:40 v/v). The cholesterol-containing organic phase was dried and dissolved in isopropyl alcohol. The tissue input was 40 mg/0.15 mL. The cholesterol concentration in the extract was determined by an automated clinical analyser (Hitachi 9000). Data are mean ± s.d. (n = 6). APP/PS1-Tg, transgenic mouse model carrying mutated human amyloid precursor protein and presenilin 1; CHOL, cholesterol; s.d., standard deviation.

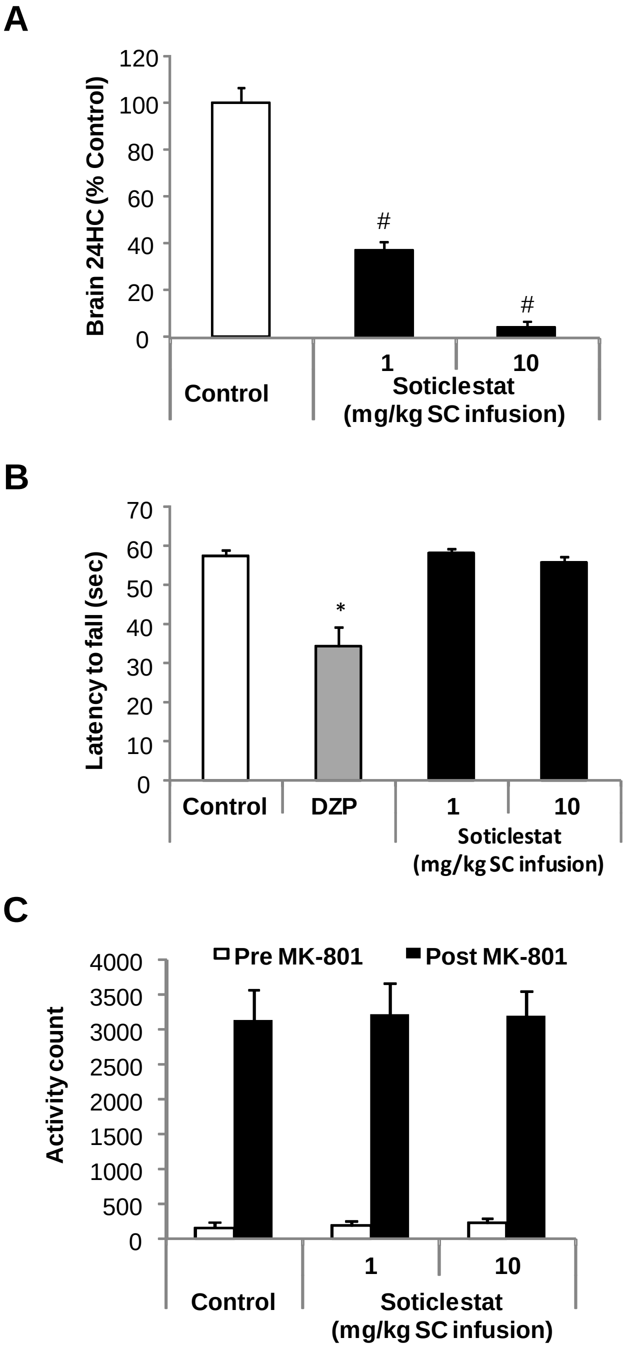

Supplementary figure S11. Impact of aggressive CH24H inhibition on normal animal behaviour by a 1-week continuous infusion of soticlestat. (**A**) Effects of soticlestat on brain 24HC. (**B**) Effects of diazepam (DZP, 2 mg/kg IP) and soticlestat on motor coordination tested in the rotarod performance. (**C**) Effects of soticlestat alone and in combination with MK-801 (0.25 mg/kg IP) on spontaneous locomotor activity. Data are mean ± s.e.m. (n = 10). $^{\#}P < 0.025$ (one-tailed Williams' test). $^{*}P < 0.05$ (Dunnett's test). 24HC, 24*S*-hydroxycholesterol; CH24H, cholesterol 24-hydroxylase; IP, intraperitoneally; SC, subcutaneous; s.e.m., standard error of measurement.

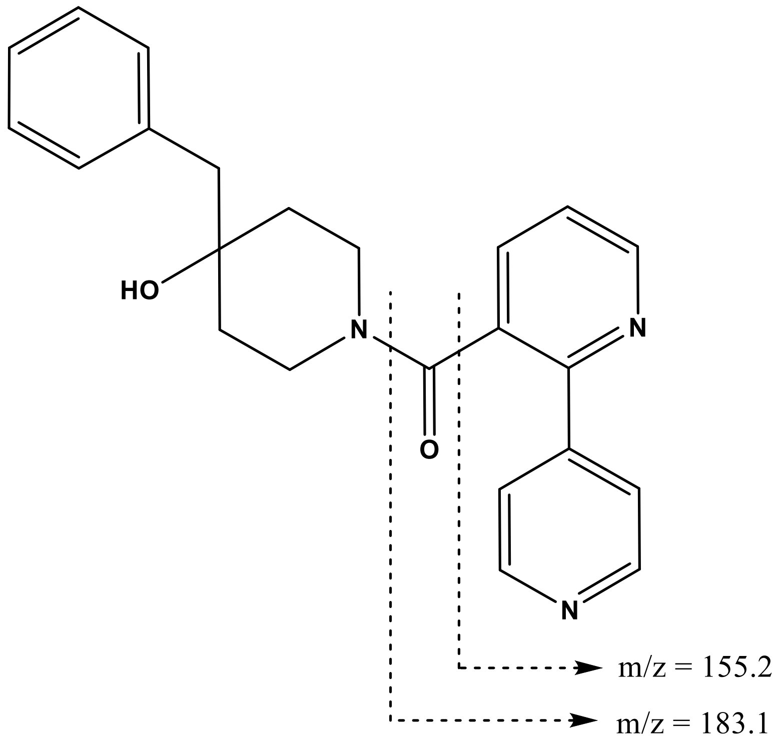

Supplementary figure S12. The chemical structure of (2,4’-bipyridin-3-yl)methanone moiety, the Q3 product ion used for quantification of soticlestat in the LC-MS/MS assay described in the Methods section of manuscript. LC-MS/MS, liquid chromatography–tandem mass spectrometry

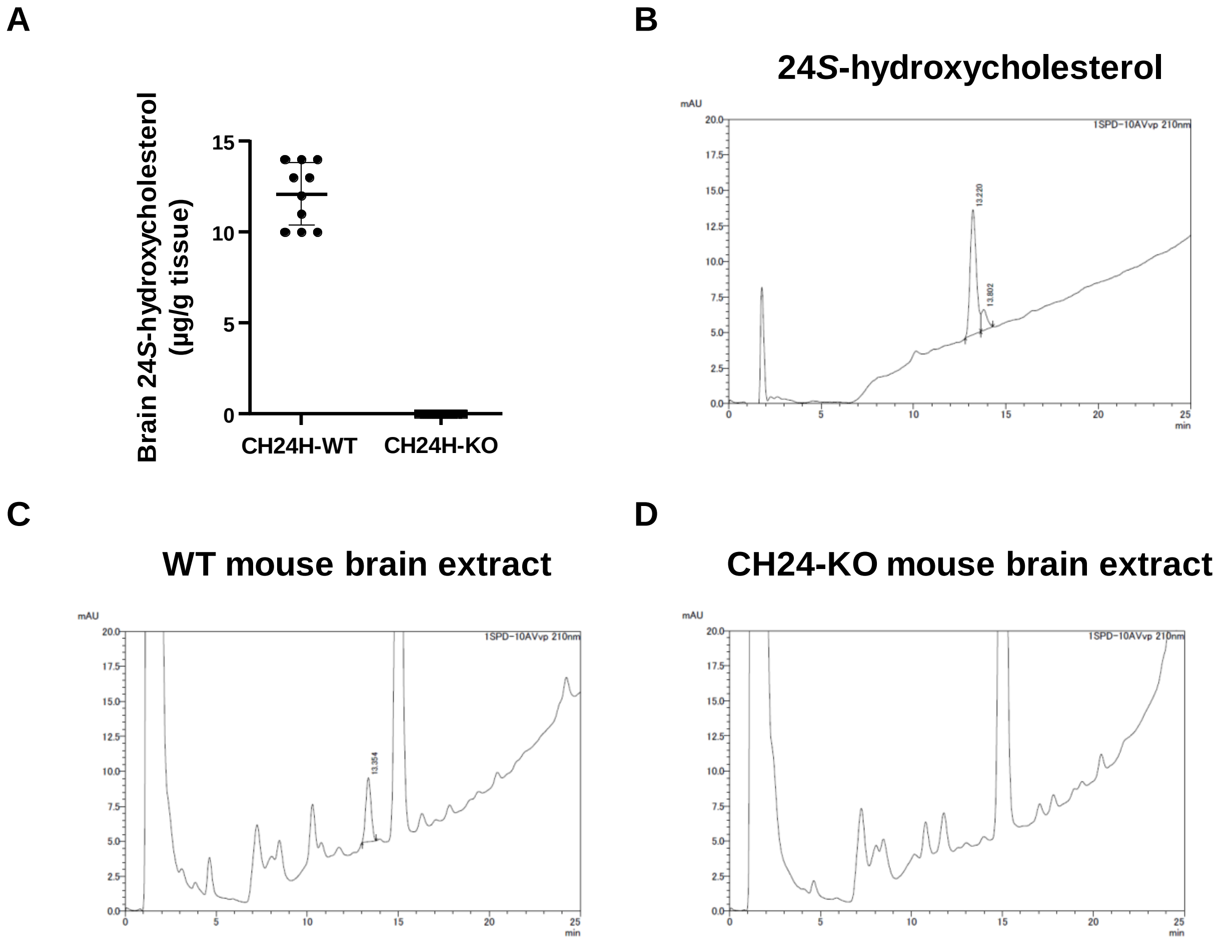

Supplementary figure S13. (**A**) Brain levels of 24*S*-hydroxycholesterol determined by LC-MS/MS following the procedure established in the previous study with some modification (J Sep Sci. 2015;38(20):3516-24). In brief, methanol homogenate of brain was prepared (500 mg/mL). Following centrifugation, the supernatant was mixed with a 4-time volume of 0.1N HCl. One mL of the mixture underwent a solid phase extraction by adding 5.5 mL of MeOH/H_2O/dichloromethane (1:4:0.5). The organic phase was dried under nitrogen stream and dissolved in 50% MeOH before injection. Data are mean ± s.d. (n = 10) (**B, C and D**) Representative UV 210 nm HPLC chromatograms of standard 24S-hydroxycholesterol, wild-type mouse brain extract and CH24H-KO mouse brain extract, respectively. The peak at a retention time of 13.2-13.3 minute was quantified as 24*S*-hydroxycholesterol. The extraction and separation followed the protocol described in the Method section. CH24H, cholesterol 24-hydroxylase; KO, knock out; LC-MS/MS, liquid chromatography–tandem mass spectrometry; WT, wild type

Assay name	*Substrate/ligand*	*Species*	*Inhibition (%)*
Adenosine A2B	[^{3}H]MRS1754	human	20
Adrenergic β1	[^{125}I]Cyanopindolol	human	-4
Adrenergic β2	[^{3}H]CGP-12177	human	6
Adrenergic β3	[^{125}I]Cyanopindolol	human	-7
Angiotensin AT2	[^{125}I]CGP-42112A	human	-10
Bradykinin B1	[^{3}H](Des-Arg^{10})-Kallidin	human	13
Cannabinoid CB1	[^{3}H]SR141716A	human	-5
Carbonic Anhydrase II	4-Nitrophenyl acetate (4-NPA)	human	6
Cholinesterase, Acetyl, ACES	Acethylthiocholine	human	0
Cyclooxygenase (COX-1)	Arachidonic acid	human	-14
Cyclooxygenase (COX-2)	Arachidonic acid	human	4
Dopamine D1	[^{3}H]SCH-23390	human	5
Dopamine D2L	[^{3}H]Spiperone	human	15
Dopamine D3	[^{3}H]Spiperone	human	5
Dopamine D4.2	[^{3}H]Spiperone	human	0
GABA B1A	[^{3}H]CGP-54626	human	6
GABA B1B	[^{3}H]CGP-54626	human	8
Histamine H1	[^{3}H]Pyrilamine	human	-2
Histamine H2	[^{125}I]Aminopotentidine	human	5
Imidazoline I2(Central)	[^{3}H]Idazoxan	human	12
MAO A	Kynuramine	human	1
MAO B	Kynuramine	human	0
Muscarinic M1	[3H]N-Methylscopolamine	human	7
Muscarinic M2	[3H]N-Methylscopolamine	human	1
Muscarinic M3	[3H]N-Methylscopolamine	human	12
Opiate κ (OP2, KOP)	[3H]Diprenorphine	human	-1

Opiate μ (OP3, MOP)	[3H]Diprenorphine	human	-1
Peptidase, Metalloproteinase, Neutral Endopeptidase	Glutaryl-Ala-Ala-Phe-4-methoxy-2-naphthylamide	human	-4
Phosphodiesterase 4	[3H]cAMP + cAMP	human	-1
Phosphodiesterase 5	[3H]cGMP + cGMP	human	-2
5-HT2B	[3H]Lysergic acid diethylamide (LSD)	human	5
Dopamine transporter	[125I]RTI-55	human	0
Norepinephrine transporter	[125I]RTI-55	human	-1
Serotonin transporter	[3H]Paroxetine	human	6
Vasopressin V1A	[125I] PhenylacetylTyr(Me)PheGlnAsnArgProArgTyr	human	-11

Supplementary table S1. Soticlestat binding affinity to known CNS drug targets at 10 µmol/L and inhibitory activity on major drug-metabolizing CYP enzymes. CNS, central nervous system; GABA, gamma-aminobutyric acid; MAO, monoamine oxidase; cAMP, cyclic adenosine monophosphate; cGMP, cyclic guanosine monophosphate; 5-HT2B, 5-Hydroxytryptamine receptor 2B.

CYP	*IC_{50} (μM)*
2C8	62
2C9	19
2D6	>100
3A4	66
1A2	>100
2C19	14

Supplementary table S2. Soticlestat inhibitory activity on major drug-metabolizing CYP enzymes. CYP, cytochrome P450; IC_{50}, half-maximal inhibitory concentration.

Characterization of chemical materials.

All commercially available reagents and solvents were used without further purification. Yields were not optimized. All reactions were monitored by thin-layer chromatography analysis on Merck Kieselgel 60 F254 plates or Fuji Silysia NH plates, or by liquid chromatography–mass spectrometry (LC-MS) analysis. Proton nuclear magnetic resonance (^{1}H NMR) spectra were recorded on a Varian Mercury-300 (300 MHz), Bruker DPX300 (300 MHz) or Bruker AVANCE III (300 MHz) spectrometer. Chemical shifts are given in parts per million downfield from tetramethylsilane (δ) as the internal standard in deuterated solvent. Chromatographic purification was performed on Purif-Pack (SI or NH, Shoko Scientific, Kanagawa, Japan). LC-MS analysis was performed on Shimadzu ultrafast LC-MS (UFLC-MS) (Prominence UFLC high-pressure gradient system/LC-MS-2020), operating in electrospray ionization (ESI) (+ or −) or atmospheric chemical ionization (+ or −) mode. The data were analysed by using the ACD/Spectrus Processor software version 2017.1.2 (Advanced Chemistry Development, Inc., Toronto, ON, Canada)

(4-benzyl-4-hydroxypiperidin-1-yl)(2-chloropyridin-3-yl) methanone. To a mixture of 2-chloronicotinic acid (1.00 g, 6.35 mmol), toluene (15 mL) and dimethyl ether (5 mL), thionyl chloride was added (0.505 mL, 6.92 mmol), and then the mixture was stirred at 90°C under N_2 for 4 h. The reaction mixture was concentrated under reduced pressure. The residue was dissolved in tetrahydrofuran (15 mL); then, triethylamine (0.965 mL, 6.92 mmol) and 4-benzyl-4-hydroxypiperidine (1.10 g, 5.77 mmol) were added, and the reaction mixture was stirred at room temperature under N_2 overnight. To the reaction mixture, saturated aqueous $NaHCO_3$ was added and extracted with ethyl acetate (EtOAc). The organic layer was separated, washed with brine, dried over Na_2SO_4 and concentrated *in vacuo*. The residue was purified by column chromatography (silica gel, EtOAc/hexane) to give title compound (1.86 g, 5.62 mmol, 97%) as a white solid. ^{1}H NMR ($CDCl_3$) δ 1.22−1.94 (5H, m), 2.80 (2H, d, *J* = 5.3 Hz), 3.08−3.60 (3H, m), 4.47−4.66 (1H, m), 7.14−7.23 (2H, m), 7.27−7.40 (4H, m), 7.55−7.71 (1H, m), 8.43 (1H, dd, *J* = 4.5, 1.9 Hz); MS (ESI) *m/z*: 331 $(M+H)^+$.

(4-benzyl-4-hydroxypiperidin-1-yl)(2,4'-bipyridin-3-yl) methanone (soticlestat). A mixture of (4-benzyl-4-hydroxypiperidin-1-yl)(2-chloropyridin-3-yl) methanone (5.00 g, 15.1 mmol), pyridine-4-boronic acid (2.23 g, 18.1 mmol), sodium carbonate (4.81 g, 45.3 mmol), $Pd(PPh_3)_4$ (0.873 g, 0.76 mmol), dimethylformamide (50 mL) and water (10 mL) was heated at 100°C

under N_2 overnight. To the reaction mixture, brine was added and extracted with EtOAc. The organic layer was separated, washed with brine, dried over Na_2SO_4 and concentrated *in vacuo*. The residue was purified by column chromatography (NH silica gel, EtOAc/hexane) to give title compound (3.42 g, 9.16 mmol, 61%) as a white solid. Crystallization from EtOAc/hexane afforded title compound as a white crystal. 1H NMR (CDCl3, mixture of rotamers) δ 0.07-1.67 (5H, m), 2.35-3.17 (5H, m), 4.41-4.60 (1H, m), 6.98-7.15 (2H, m), 7.22-7.32 (3H, m), 7.41 (1H, dd, J = 7.5, 5.0 Hz), 7.61 (1H, d, J = 4.2 Hz), 7.70-7.83 (2H, m), 8.60-8.82 (3H, m); 13C NMR (CDCl3, mixture of rotamers) δ 35.6, 36.0, 36.2, 36.7, 37.6, 42.5, 43.0, 49.1, 49.2, 68.8, 69.0, 122.8, 123.5, 126.9, 128.4, 130.3, 131.5, 131.8, 135.4, 135.6, 136.0, 136.1, 146.3, 150.0, 150.3, 151.6, 152.2, 167.2, 167.4; MS (ESI) m/z: 374 (M+H)+.

Analysis of 24HC ratio.

The effect of soticlestat, a cholesterol 24-hydroxylase inhibitor, was evaluated by measuring the reduction of brain 24*S*-hydroxycholesterol (24HC). The 24HC reduction ratios in mice brains were calculated as the ratio of the ultraviolet (UV) peak area of brain 24HC of a test animal compared with the mean value of vehicle animals. The percentage values of the brain 24HC reduction were calculated as shown below.

Brain 24HC ratio (%) = (UV peak area of brain 24HC in a test animal/mean value of UV peak area of brain 24HC in vehicle animals) × 100

Given that the data were normalized by the control levels, correlation analyses excluded the data of the control group.

Rotarod test.

Motor coordination was assessed with the rotarod test following protocols published elsewhere. The drum was rotated at the speed of six rounds per minute. After 2 min of a training session, mice underwent a 60 s testing session repeated three times. The latency to fall off the rotarod was recorded, and motor coordination was evaluated as the mean latency of the three trials.

Spontaneous locomotor activity.

The effects of soticlestat on locomotor activity were assessed following its subcutaneous infusion treatment for 2 weeks in mice. Mice treated with either vehicle or soticlestat were

transferred to a behavioural box (length × width × height: 36.0 × 22.0 × 13.5 cm) 120 min before the beginning of the dark phase for habituation. Activity counts were measured as the number of infrared beam breaks at 1 min intervals (MDC system, Brain Science Idea Co., Ltd., Osaka, Japan). Cumulative counts were calculated for 120 min before and after injection of MK-801 (0.25 mg/kg).
